# BioModels: expanding horizons to include more modelling approaches and formats

**DOI:** 10.1093/nar/gkx1023

**Published:** 2017-11-02

**Authors:** Mihai Glont, Tung V N Nguyen, Martin Graesslin, Robert Hälke, Raza Ali, Jochen Schramm, Sarala M Wimalaratne, Varun B Kothamachu, Nicolas Rodriguez, Maciej J Swat, Jurgen Eils, Roland Eils, Camille Laibe, Rahuman S Malik-Sheriff, Vijayalakshmi Chelliah, Nicolas Le Novère, Henning Hermjakob

**Affiliations:** European Molecular Biology Laboratory, European Bioinformatics Institute (EMBL-EBI), Wellcome Genome Campus, Hinxton, Cambridge CB10 1SD, UK; Department of Bioinformatics and Functional Genomics, Biomedical Computer Vision Group, University of Heidelberg, BioQuant, IPMB and DKFZ Heidelberg, Im Neuenheimer Feld 267, 69120 Heidelberg, Germany; University of Rostock, Rostock, Germany; Babraham Institute, Cambridge CB22 3AT, UK

## Abstract

BioModels serves as a central repository of mathematical models representing biological processes. It offers a platform to make mathematical models easily shareable across the systems modelling community, thereby supporting model reuse. To facilitate hosting a broader range of model formats derived from diverse modelling approaches and tools, a new infrastructure for BioModels has been developed that is available at http://www.ebi.ac.uk/biomodels. This new system allows submitting and sharing of a wide range of models with improved support for formats other than SBML. It also offers a version-control backed environment in which authors and curators can work collaboratively to curate models. This article summarises the features available in the current system and discusses the potential benefit they offer to the users over the previous system. In summary, the new portal broadens the scope of models accepted in BioModels and supports collaborative model curation which is crucial for model reproducibility and sharing.

## INTRODUCTION

Mathematical models representing biological processes and computational simulations have been shown to provide mechanistic insight into the functional properties of biological systems. BioModels (1) emerged in 2005 as a response to the needs of the systems biology community for a resource that facilitates the exchange, reuse and repurposing of the models. Since its inception, BioModels’ content has been steadily increasing, making it a central portal that provides reproducible, high-quality, freely accessible models published in the scientific literature. BioModels hosts over 8400 models from the scientific literature submitted by authors as well as internal and external BioModels curators. This includes a recent submission of 6750 patient-specific genome-scale metabolic models derived from tumour samples of individual patients (2). Where possible, models submitted to BioModels are manually curated to reproduce the simulation figures in the reference publication and are semantically enriched using cross references to external database resources and ontologies. BioModels also hosts over 140 000 models generated from biological pathways produced by the Path2Models project (3). All hosted models are freely available to access and download through BioModels, either individually from our website (http://www.ebi.ac.uk/biomodels) or as release archives from our FTP server (ftp://ftp.ebi.ac.uk/pub/databases/biomodels/), under Creative Commons (CC0) licence. Thereby BioModels allows modellers to submit and share their models with the broader scientific community.

Mathematical modelling of biological systems has become increasing popular over past five decades and diverse modelling approaches have evolved over this period. To cater for the growing need to host models of different approaches and formats, a new infrastructure has been developed and made available at www.ebi.ac.uk/biomodels. The new application is based on **JU**st a **M**odel **M**anagement **P**latform (JUMMP) (https://bitbucket.org/jummp/jummp), a free, open source, solution for managing mathematical models. One of the landmark improvements brought by this upgrade is the provision of a flexible platform that allows submitting and hosting of models in diverse encoding formats. Unlike our previous platform that primarily supports SBML (4) and CellML (5), the current one can support models built using different modelling approaches and softwares. Additional features include a powerful faceted search engine and enhanced provision for collaborative curation.

## MULTIFORMAT MODEL SUBMISSION

BioModels aims to make open-access sharing of high-quality physiologically and pharmaceutically relevant models from scientific literature easy. Its endorsement for community standards used to encode models and associated metadata, combined with its curation efforts have established it as one of the most widely used repositories of its kind (6). The combination of these factors facilitates the exchange, reuse and repurposing of the models hosted therein.

**Figure 1.**
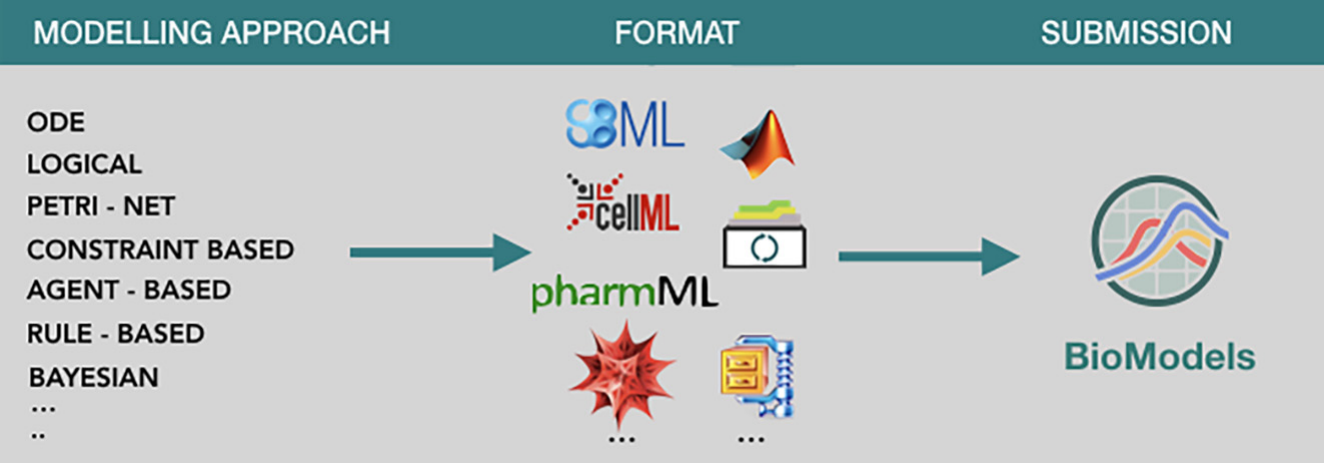
Shows some of the modelling approaches and formats that BioModels supports with its current JUMMP-based infrastructure. In addition to model files, the current platform also allows the submission of other supporting model related files.

Key parts of the functionality in the previous system (1), including the curation pipeline and the model display page, are contingent on the model being encoded in standard formats including SBML or CellML. For submissions in other formats curators resort to creating a skeletal SBML representation that references the original model. The new infrastructure removes this technical hindrance, allowing authors to submit models encoded in the format of their choice including Python, Mathematica or Matlab SimBiology, and regardless of the modelling approach employed (Figure 1). To foster interoperability and model reuse, the new system offers enhanced technical support for open formats such as SBML, PharmML (7) or COMBINE Archive (8). This expansion of scope for submissions offers more modelling communities the ability to leverage BioModels for dissemination, exchange and reuse of the models that they develop.

The new submission process is organised as a series of steps, allowing contributors to upload their models and any additional relevant information. The outcome of a modelling exercise comprises not just model files, but also data, execution scripts and plots. The new platform streamlines uploading all this information with a single click (for example, a COMBINE archive), and it is also possible to provide individual files one by one. The submission details can be reviewed and amended prior to completing the submission, minimising the risk of omissions. Models submitted to BioModels are assigned a perennial submission identifier that can be used as a means of referencing the model in publications as well as retrieving it.

## COLLABORATIVE CURATION

To ensure reproducibility and facilitate reuse, models submitted to BioModels are curated and annotated by the curation team, often with help from the authors. Models are independently verified to reproduce the simulation results in the reference publication. Furthermore, to unambiguously identify model entities, they are linked to publicly available external resources used in Life Sciences such as online databases, controlled vocabularies or ontologies by means of identifiers.org (9) Uniform Resource Identifiers. Although manual curation and semantic enrichment make a model more suitable for dissemination, reuse and repurposing, these processes are laborious and time consuming. The new system provides several features for streamlining them.

In the previous infrastructure, submitted model files could only be updated by curators at the request of authors. The current version allows authors to perform a range of actions on the models from submission to curation.

### Updating models

Users may add, modify or remove files associated with a submission, or update the publication details and annotations as necessary. The changes are organised as a series of incremental snapshots or revisions of the model that are immutable. Previous revisions can be accessed and downloaded even after newer changes have been introduced. This development history workflow is similar to flexible version control systems like Git (http://www.git-scm.com) or Subversion (https://subversion.apache.org), but without the associated technical proficiency requirements.

### Managing model access rights

**Figure 2.**
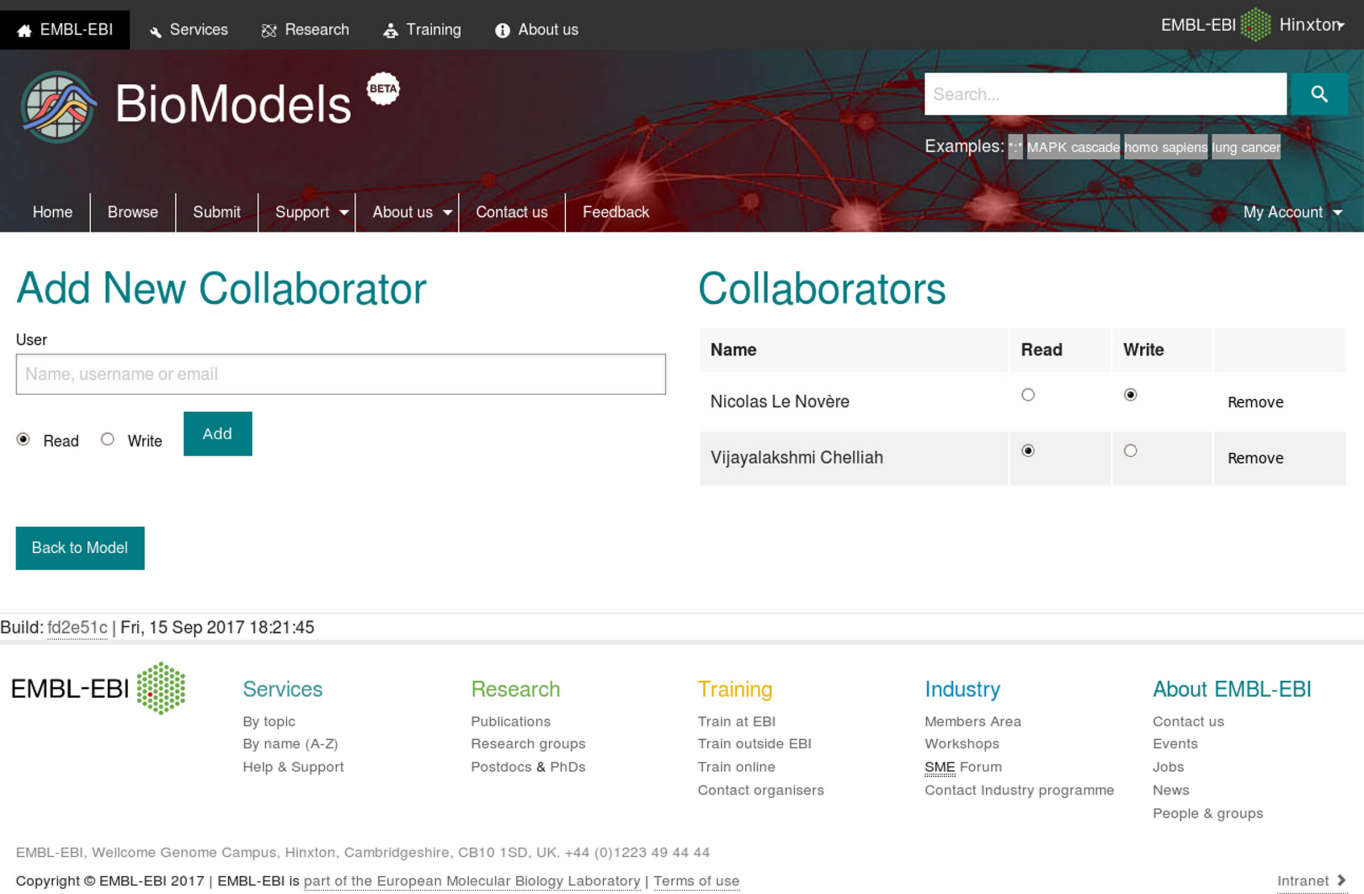
Models can be shared with specific users or teams. Submitters can search for collaborators through the text field on the left-hand-side of the page which provides autocompletion and can choose whether to grant them read-only or read-write permissions. Existing collaborators can be managed using the controls on the right-hand-side of the page.

**Figure 3.**
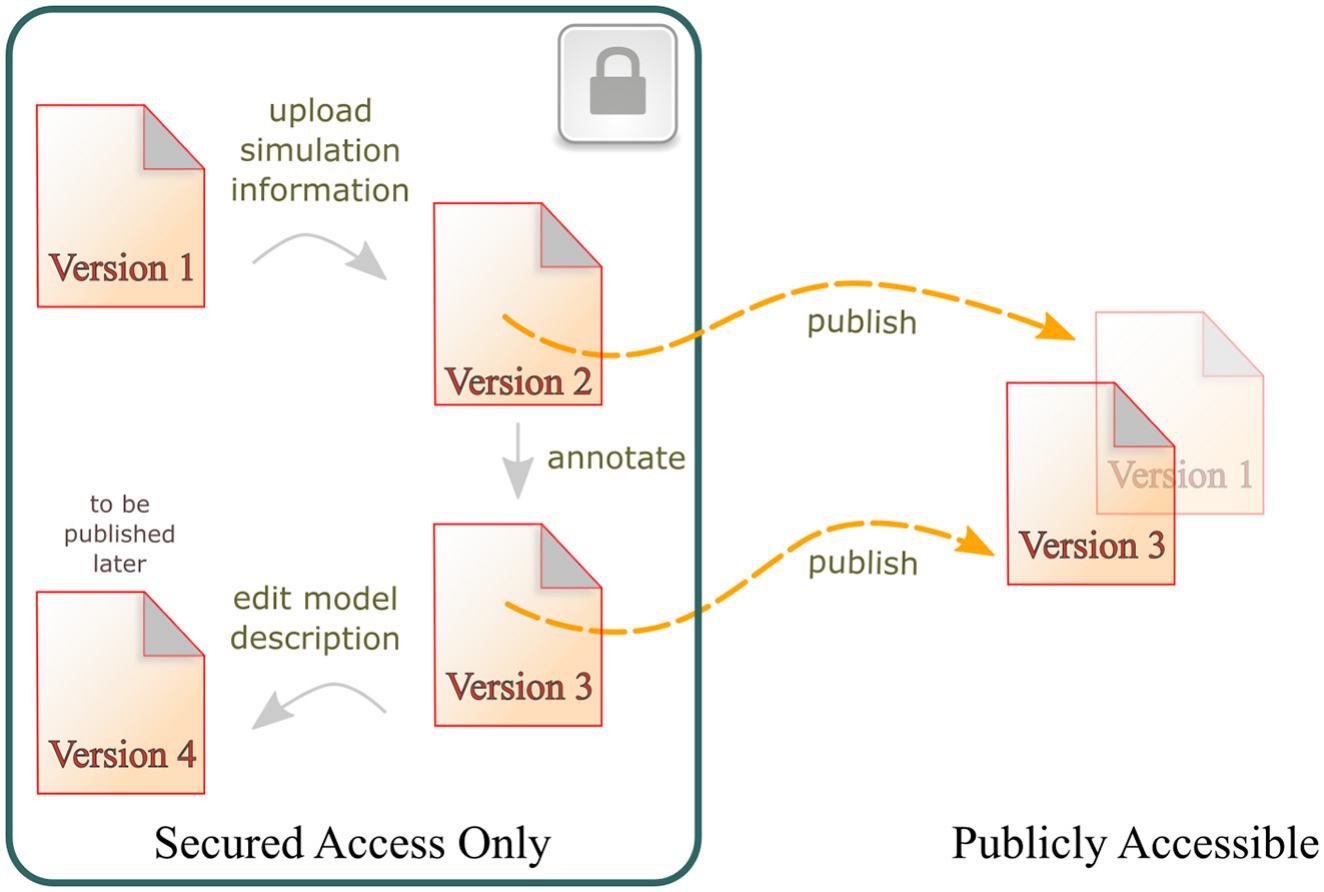
Representation of a sample life cycle for a model in the new portal of BioModels. The full modification history of the model, captured as a series of revisions, is available to the submitter. Only revisions that have been published are publicly available. Submitters can continue to revise models privately even after publishing them.

Model development is inherently a collaborative effort, bringing together people with different backgrounds and even from different organisations. Often models undergo several refinements before being fit for purpose. In recognition of this, model revisions in the new version of the service are private by default. Submitters can share a model with individual users or with teams of collaborators, choosing to grant them read-write or read-only access (Figure 2). Specific revisions can be made publicly available by a BioModels curator once they meet the required criteria (Figure 3). Once submitted, models cannot be expunged from the system. They can be archived, meaning that they will be excluded from search results, but can still be accessed. Models with at least one public revision cannot be archived, ensuring that they remain available for the foreseeable future.

### Content update notifications

Notifications help BioModels users stay informed of changes to the models they are working on. Users can receive updates by email and/or through the website. The system sends notifications to all collaborators when a model is updated, archived or when a version of the model is published; it also notifies users when they gain access to a model or when a model is shared with a new contributor.The support for uploading models in any format, coupled with the sharing and access control capabilities of the current system make it possible for modellers that are not familiar with community standards for representing models to encode their existing models into these open formats with the help of the community, to the benefit of both parties.

Reliable, well-annotated models in standard formats are more likely to be reused by others. The new infrastructure recognises the need for this collaboration and helps submitters take a more proactive stance in the model curation process. Moreover, submitters can start using BioModels much earlier in the model development phase thanks to the versioning capabilities of the current system.

## MODEL SEARCH AND RETRIEVAL

**Figure 4.**
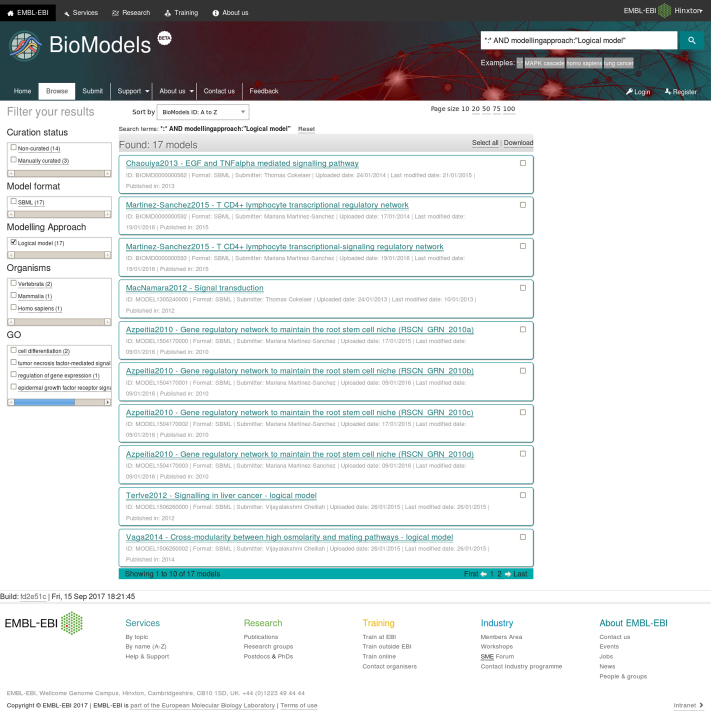
Screenshot of the search results for logical models. The left-hand-side column allows users to progressively filter their models of interest. Long search results are broken into separate chunks (pages) of customisable size that can be accessed independently. Different sorting criteria are available. A subset of the search results can be selected for bulk download.

An effective search is critical to any repository that seeks to encourage model dissemination and reuse. The upgraded search system, based on the Omics Discovery Index (10) and the EBI search engine (11), retains the in-depth capabilities of the previous system whilst offering a streamlined experience for end users in terms of speed and ease of use. Unlike other databases that only allow searching within metadata, BioModels leverages both the content of the model files and the semantic enrichment for searching. Search results can be filtered based on various model characteristics including curation status, formats, modelling approaches, organism or disease (Figure 4). Once the search results have been narrowed down sufficiently, the new version of the service lets users download the matching models as an archive with the click of a button. Search results can be sorted based on author name, publication year, model name or BioModels identifier or downloaded as an archive.

The previous BioModels platform offers a very detailed representation of models encoded in SBML organised in tabs covering information about the model entities, parameters as well as mathematical relationships between entities along with associated metadata. This level of detail poses two important challenges: on the one hand, it is tied to SBML, making it difficult to offer a consistent user experience across different model formats; on the other hand, it is not flexible enough to keep abreast the cornucopia of SBML Level 3 packages being developed. The current version of the service tackles these issues by grouping tabs based on whether their contents are dependent on the format used to encode the model to be displayed.

**Figure 5.**
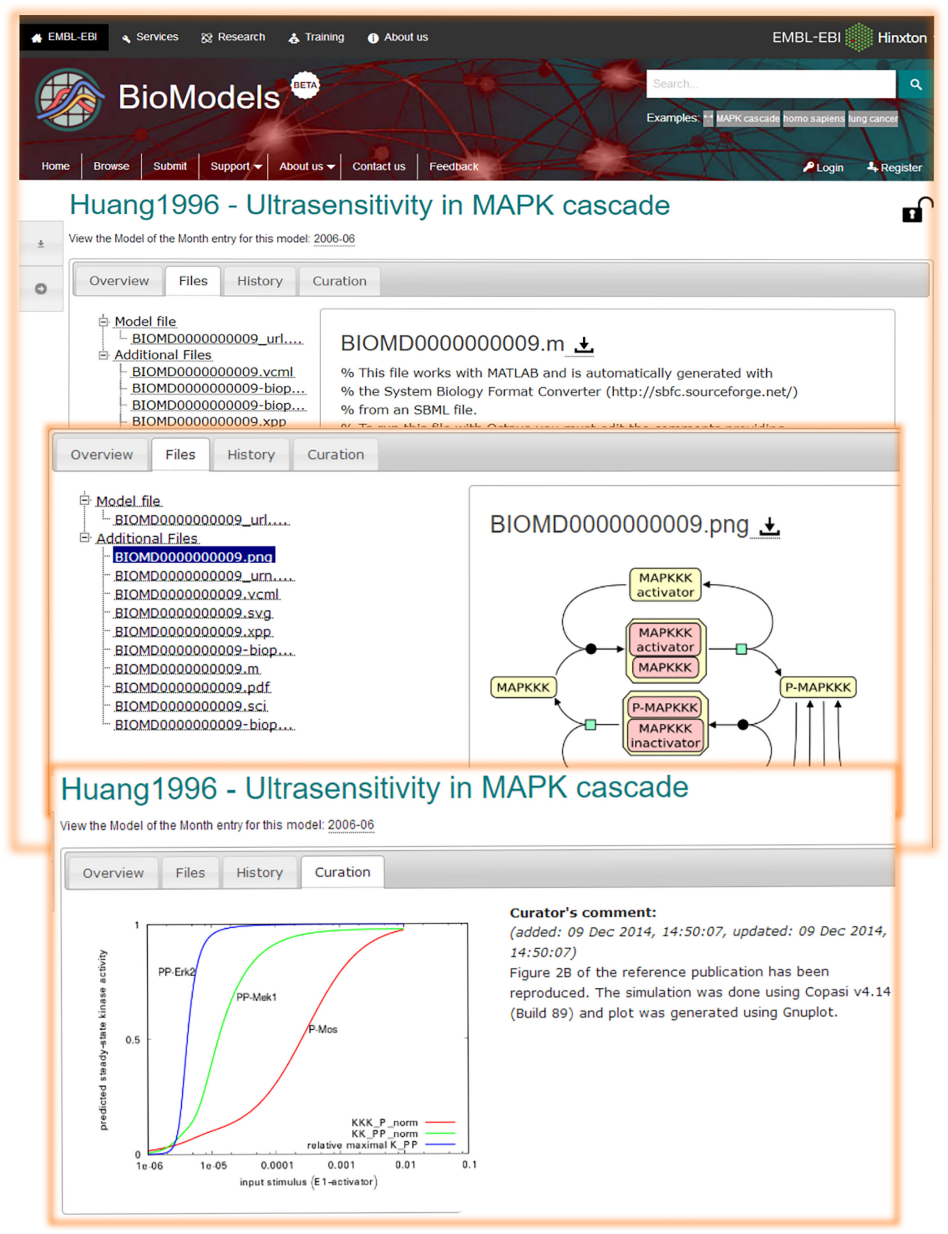
The model display page offers a high-level summary of the model organised arranged into a series of tabs. The first three tabs are always shown, whilst subsequent ones depend on the model’s curation status and encoding format.

The first of the format-independent tabs is entitled overview and it aims to give a non-technical summary of the model. Next, the Files tab allows users to preview and download the contents of the individual files that are part of the submission. The History tab offers access to all the previous revisions of the model displayed. The contents of this tab for the same model may vary between users depending on their respective access rights. In addition, for models that have been curated, a fourth tab displays the simulation figure produced in the curation process along with any comments concerning reproducibility (Figure 5). This set of tabs may be complemented by format-specific tabs that aid the comprehension of the model displayed and the evaluation of its relevance to the user’s needs.

External tools use the web services exposed by the earlier BioModels infrastructure (12) to allow their users to search and retrieve relevant BioModels content remotely. Although the functionality previously offered is quite effective, allowing tools to easily fetch models based on their curation status, accession or cross reference from sources such as NCBI Taxonomy, Uniprot, Gene Ontology, ChEBI or Reactome, the SOAP-based mechanism of interaction is verbose and carries significant technical overheads. The current system lowers the technical barriers for programmatically accessing BioModels content through the introduction of a new, easy-to-use RESTful application programming interface (API). In addition, the updated API can offer both JSON and XML responses, thus any mainstream programming language as well as tools like curl (http://curl.haxx.se) and wget (https://www.gnu.org/software/wget) can be used to retrieve and process the information available.

## CONCLUSION

The modelling landscape is constantly evolving and BioModels is adapting. The advent of encoding standards has lead to the creation of ever-larger models containing tens of thousands of reactions (arXiv: https://arxiv.org/abs/1311.5696), which put a strain on conventional repositories. The new system, developed in response to the current and upcoming challenges facing model repositories, offers a solid foundation on which to make BioModels better for existing users and submitters, but also to attract new ones. We recognise that continuity is very important, particularly for the plethora of tools that rely on the programming interface of the previous system. In this respect, the latter will remain functional for some time in parallel with the updated platform, where models should be submitted. BioModels can be cited in accordance with the information given on our citation page (https://www.ebi.ac.uk/biomodels-main/citation#biomodels).

BioModels now encompasses significant new features that will aid productivity and allow a more diverse range of models to be submitted. Although presently the new system contains the published models of BioModels that are primarily accessed by our users, the Path2Models branch will become available in due course.

Whilst the features presented herein unlock a world of opportunities for current and prospective BioModels submitters and users, we are actively working on bringing more new features and enhancements to the new platform. These include making the complete BioModels dataset available, developing enhanced previews for models in SBML, but also consolidating the curation capabilities of the resource to cover more modelling approaches, such as constraint-based models, and a lightweight annotation editor.
